# Template-based automation of treatment planning in advanced radiotherapy: a comprehensive dosimetric and clinical evaluation

**DOI:** 10.1038/s41598-019-56966-y

**Published:** 2020-01-16

**Authors:** Savino Cilla, Anna Ianiro, Carmela Romano, Francesco Deodato, Gabriella Macchia, Milly Buwenge, Nicola Dinapoli, Luca Boldrini, Alessio G. Morganti, Vincenzo Valentini

**Affiliations:** 1 Medical Physics Unit, Fondazione di Ricerca e Cura Giovanni Paolo II - Università Cattolica del Sacro Cuore, Campobasso, Italy; 2 Radiation Oncology Unit, Fondazione di Ricerca e Cura Giovanni Paolo II - Università Cattolica del Sacro Cuore, Campobasso, Italy; 3 Radiation Oncology Department, DIMES Università di Bologna - Ospedale S.Orsola Malpighi, Bologna, Italy; 4 Dipartimento di Diagnostica per Immagini, Radioterapia Oncologica ed Ematologia, UOC Radioterapia Oncologica, Fondazione Policlinico Universitario “A. Gemelli” IRCCS, Roma, Italy

**Keywords:** Endometrial cancer, Prostate cancer, Radiotherapy, Head and neck cancer

## Abstract

Despite the recent advanced developments in radiation therapy planning, treatment planning for head-neck and pelvic cancers remains challenging due to large concave target volumes, multiple dose prescriptions and numerous organs at risk close to targets. Inter-institutional studies highlighted that plan quality strongly depends on planner experience and skills. Automated optimization of planning procedure may improve plan quality and best practice. We performed a comprehensive dosimetric and clinical evaluation of the Pinnacle^3^ AutoPlanning engine, comparing automatically generated plans (AP) with the historically clinically accepted manually-generated ones (MP). Thirty-six patients (12 for each of the following anatomical sites: head-neck, high-risk prostate and endometrial cancer) were re-planned with the AutoPlanning engine. Planning and optimization workflow was developed to automatically generate “dual-arc” VMAT plans with simultaneously integrated boost. Various dose and dose-volume parameters were used to build three metrics able to supply a global Plan Quality Index evaluation in terms of dose conformity indexes, targets coverage and sparing of critical organs. All plans were scored in a blinded clinical evaluation by two senior radiation oncologists. Dose accuracy was validated using the PTW Octavius-4D phantom together with the 1500 2D-array. Autoplanning was able to produce high-quality clinically acceptable plans in all cases. The main benefit of Autoplanning strategy was the improvement of overall treatment quality due to significant increased dose conformity and reduction of integral dose by 6–10%, keeping similar targets coverage. Overall planning time was reduced to 60–80 minutes, about a third of time needed for manual planning. In 94% of clinical evaluations, the AP plans scored equal or better to MP plans. Despite the increased fluence modulation, dose measurements reported an optimal agreement with dose calculations with a γ-pass-rate greater than 95% for 3%(global)-2 mm criteria. Autoplanning engine is an effective device enabling the generation of VMAT high quality treatment plans according to institutional specific planning protocols.

## Introduction

In the past two decades, significant advances in planning and delivery of radiotherapy have been accomplished. In particular, the developments of new optimization algorithms in treatment planning systems (TPS) and the new capabilities of linear accelerators led to the implementation of intensity-modulated techniques (IMRT), able to improve dose distributions and conformity, reducing the irradiation to normal tissues and thereby minimising the risk of toxicity. Volumetric modulated arc therapy (VMAT), a complex technique able to deliver radiation beams in continuous sweeping arc around the patient, had a widespread diffusion in the last years^1^. This technology provided similar plan quality with respect to fixed-field IMRT in terms of target dose conformality and minimal exposure to surrounding healthy tissues but with a large reduction in treatment time, potentially reducing the risk of intrafraction motion and improving patient comfort.

Despite the advanced developments in radiation therapy planning over the past years, complex treatment planning with VMAT or IMRT still remains challenging in order to achieve clinically acceptable plans. The manual planning process needed for these advanced techniques usually requires several trial-and-error optimization processes in which the continuous manual tuning of dosimetric objectives and weights translates in overall quality of plans strongly depending on planner experience^2^.

Various algorithms have been proposed for an automated optimization of the planning procedure and the search for the optimal patient plan in order to improve best practice. In particular, fully automated IMRT and VMAT plans have been successfully generated for clinical application using new commercial solutions. Knowledge-based planning (KBP) algorithms use a mathematical model, built and trained from a large number of previous optimal plans, to estimate the dose distribution and dose-volume histograms (DVH) for any new patient. This strategy has been implemented in the Varian Eclipse treatment planning system (TPS) as Rapidplan module (Varian Medical Systems, Palo Alto, Ca) and has been recently tested in different anatomical sites reporting an overall improving of treatment plans quality^3–8^ with respect to conventional manual planning. Multi-criteria optimization (MCO) provides an alternative optimization workflow by generating a set of Pareto-optimal treatment plans regarding user-specified priorities and objectives (eg. plans for which no objectives can be improved without impairing another one)^9^. The planner can then navigate on the so-called Pareto surface from one plan to another to balance between clinical trade-offs. This strategy has been implemented in the Raystation TPS (Raysearch, Stockholm, Sweden) and in the Erasmus-Icycle algorithm developed at Erasmus MC-Cancer Institute in Rotterdam, reporting successful application in specific sites^10–16^. Another strategy for planning automation is the template-based planning optimization process. This algorithm, implemented in the Autoplanning module of Pinnacle^3^ TPS (Philips Medical Systems, Fitchburg, WI) does not require any prior database of successful plans nor model training but uses an iterative approach of progressive optimization that mimic all the steps of experienced and skilled planners^17^. Autoplanning automates the inverse planning process using a so-called “Technique”, i.e. a template of parameters including beam setup, dose prescriptions and planning objectives that can be customized for each treatment protocol and tumor site. Then, the Autoplanning engine applies the Technique to iteratively optimize planning parameters to best meet the desired planning goals; different kinds of dummy structures are automatically generated and new objectives are added to the planning goals list in order to achieve better organs-at-risk (OARs) sparing and target uniformity and conformity by reducing cold/hot spots and managing the dose fall-off outside the targets. Autoplanning has been recently tested in a few publications for VMAT treatments of prostate cases^18^, head-neck tumours^19^, esophageal cancer^20^ and for the extracranial stereotactic treatment of liver^21^ and lung^22^ metastasis, reporting in all cases promising results. All the aforementioned studies provided useful investigations of Autoplanning but were limited to single cancer sites.

The aim of the present study was to provide a more comprehensive evaluation of the Autoplanning potential including several challenging sites, normally treated in the clinical routine, as bilateral head-neck tumours, high-risk prostate cancer and endometrial cancer. In all these sites, several large irregular-shaped targets volumes, multiple dose prescription levels and the large number of complex anatomical organs at risk close or overlapped to targets represent a major challenge for the generation of optimal quality plans. VMAT plans for these cancer cases should then require highly conformal dose distributions and steep dose-gradient between targets and the many critical organs-at-risk.

Based on the recent literature data, in this study we hypothesized that automated radiotherapy treatment planning has the potential to increase consistency, improve plan quality and reduce workload for all routinely challenging treatments. The automated plans were then compared with the clinically accepted VMAT treatment plans manually generated by experienced medical physicists.

## Material and Methods

### Ethics statement

This study was approved by the Institutional Review Board of the Fondazione di Ricerca e Cura Giovanni Paolo II in Campobasso, Italy. Because this was not a treatment-based but a retrospective dosimetric planning study, our institutional review board waived the need for written informed consent from the participants. The patient information was anonymized and de-identified to protect patient confidentiality. All the methods described here were performed in accordance with the relevant guidelines and regulations.

### Patient population and volumes definition

Three different complex anatomic sites are presented to demonstrate the potential and challenges of automated template-based VMAT planning with respect to conventional manual planning. A total of 36 patients previously treated with SIB-VMAT were randomly selected, 12 patients for each of the following pathologies: bilateral head-neck, high-risk prostate and endometrial cancer. All patients underwent a simulation computed tomography (CT) scan (3-mm slice thickness); MRI-imaging for endometrial and prostate cases and PET-CT imaging for head-neck cases were then co-registered with the simulation CT for accurate volume delineation.

### Head-and-neck cancer

The primary tumour and nodes showing metabolic activity at 18F PET-CT scan were defined as clinical target volume 1 (CTV1). CTV2 and CTV3 were defined as lymph nodes with high-risk and low-risk of occult metastases, respectively. Lymph nodal regions were outlined according to Grégoire *et al*. guidelines^23^. Corresponding planning target volumes (PTVs) were obtained by adding an isotropic 4-mm margin to CTVs. All PTVs were simultaneously irradiated over 30 daily fractions according to the SIB technique. Doses of 67.5 Gy (2.25 Gy/fraction), 60.0 Gy (2.0 Gy/fraction), and 55.5 Gy (1.85 Gy/fraction) were prescribed to the PTV1, PTV2, and PTV3, respectively. A planning organ-at-risk volume (PRV) was defined for serial OARs (spinal cord, brainstem, optic chiasm and optic nerves) isotropically expanding the corresponding OAR by 5 mm. For all serial PRV_OARs, the dose to 0.035 cc was considered as maximal dose.

### High-risk prostate cancer

The prostate plus 5-mm periprostate tissue and 5 to 20 mm of caudal seminal vesicles based on risk category^24^ were defined as CTV1. CTV2 included the obturator, internal and external iliac, and presacral lymph nodes. PTV2 was obtained by adding an isotropic 8-mm margin to the CTV2; PTV1 was obtained by adding a 8-mm margin in all directions to the CTV1, except posteriorly where a 6-mm margin was given. The PTVs were simultaneously irradiated over 25 daily fractions prescribing 65.0 Gy (2.6 Gy/fraction) and 45.0 Gy (1.8 Gy/fraction) to PTV1 and PTV2, respectively. Main OARs were considered the rectum, the bladder, the small bowel and the femurs.

### Endometrial cancer

The PTV1 consisted of the upper two thirds of vagina plus resection lines in the parametria (CTV1) with a 8 mm margin. CTV2 included the obturator, external iliac, internal iliac and presacral nodes. Corresponding PTV2 was obtained adding 8 mm margin. The PTVs were simultaneously irradiated over 25 daily fractions with a dose of 60.0 Gy (2.4 Gy/fraction) and 45.0 Gy (1.8 Gy/fraction) to PTV1 and PTV2, respectively. Main OARs were the rectum, the bladder, the small bowel and the femurs.

### Manual and auto- VMAT planning

Manual VMAT plans were generated with “dual-arc” feature using the inverse optimization process previously described in more details^25^ for coplanar 6–10 MV photon beams of an Elekta VersaHD linac (Elekta Ltd., Crawley, UK). A full gantry rotation was described by a sequence of 90 control points, i.e. one every 4°. Collimator was set at 10° to minimize the tongue-and-groove cumulative effect. For the three anatomical sites, treatment planning was performed accounting to the clinical objectives reported in Table 1.

**Table 1:** Clinical objectives for treatment planning at Fondazione di Ricerca e Cura “Giovanni Paolo II”.

ROI	Goal type	Dose	Volume
Head-neck cancer			
PTV1, PTV2 and PTV3	DVH	95%	98%
	DVH	98%	95%
	DVH	107%	5%
Parotids	Mean dose	25 Gy	
Spinal cord	Max dose	45 Gy	
PRV Spinal cord	Max dose	50 Gy	
Brainstem	Max dose	50 Gy	
PRV Brainstem	Max dose	54 Gy	
Optic chiasm	Max dose	50 Gy	
PRV Optic chiasm	Max dose	55 Gy	
Retina	Max dose	40 Gy	
Lens	Max dose	5 Gy	
Optic nerves	Max dose	55 Gy	
Pharyngeal constr. muscles	Mean dose	55 Gy	
Cochleas	Mean dose	45 Gy	
Mandible	DVH	68 Gy	2%
High-risk prostate and endometrial cancer			
PTV1 and PTV2	DVH	95%	98%
	DVH	98%	95%
	DVH	107%	5%
Rectum	DVH	65 Gy	25%
	DVH	60 Gy	35%
	DVH	50 Gy	50%
Bladder	DVH	70 Gy	35%
	DVH	65 Gy	50%
Femurs	Max dose	50 Gy	
Small Bowel	DVH	15 Gy	120cc

For each PTV the doses are expressed as percentage of dose prescription.

Automated plans were created for each patient using the Autoplanning module implemented in the version 16.0 of Pinnacle^3^ TPS. For each anatomical site, the Technique was created based on the same beam parameters, dose prescription and clinical objectives adopted for manual plans. During the optimization process, the AP engine automatically generates several dummy structures including: (a) rings around the PTVs to manage the dose fall-off, (b) residual targets structures where overlaps between no-compromised OARs are removed, (c) residual OARs structures where overlaps between targets are removed, (d) body structures used to control body dose and (e) hot-spot and cold-spot structures to manage target dose uniformity. New objectives are then automatically added to the aforementioned structures in order to achieve better OARs sparing and target uniformity and conformity. This process is iteratively performed during multiple optimization loops by adjusting the optimization parameters in order to continually spare the OARs without compromising the target coverage, i.e. mimicking what a manual experienced planner would usually do. Five patients for each site, not included in the present series, have been used to create and tweak the initial Techniques in order to generate plans fulfilling the clinical objectives. For both MP and AP plans, dose calculations were performed using the Pinnacle^3^ collapsed cone convolution dose calculation algorithm with a dose grid resolution of 2 mm.

**Figure 1:**
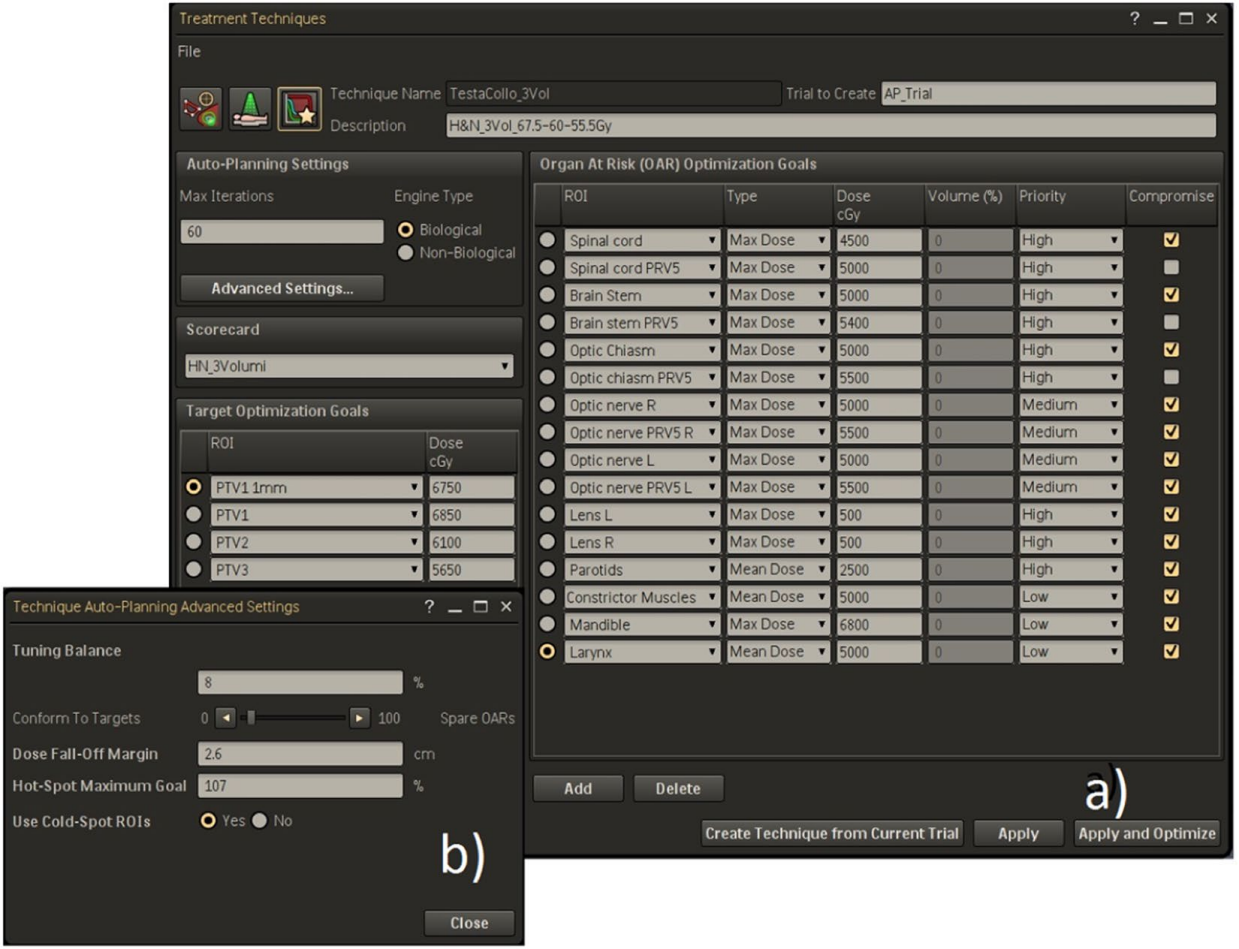
(**a**) AP setup template for head-neck cases; (**b**) advanced settings template.

An example of Technique used for head-neck cases optimization is presented in Fig. 1. The objectives for the PTVs are only defined by numbers close to prescription doses (in our experience we chose as target goals the prescription doses plus 1 Gy, so as to avoid possible under dosage in PTVs boundary). The OARs objectives include maximum dose, mean dose and dose-volume histogram points; they can have three different priority levels (high, medium and low) and can be set compromised or uncompromised. These last choice is applicable when there is an overlap between PTVs and OARs; in the case of compromise option, the PTVs owns the overlapping voxels for the benefit of target coverage. Only for serial OARs, as spinal cord and brainstem, we chose to have higher priority than target volumes. In the advanced settings template (Fig. 1b), the planner can set up: (a) the tuning balance (i.e. the balance between target dose conformity and OARs sparing), (b) the dose fall-off margin (i.e. the distance across which the dose should decrease from 80% to 20% in an automatically generated tuning ring structure around the PTVs) and (c) the Cold-Spot ROI (i.e. the identification of cold regions inside the PTVs and the automatic creation of new tuning volumes and relative dose objectives to increase dose in the last optimization loops).

For head-neck cases, a manual fine tuning of 15 minutes at the end of the automated optimization process is usually needed in order to further lower the dose to serial OARs (eg. spinal cord).

Based on the Quantitative Analyses of Normal Tissue Effects in the Clinic (QUANTEC) guidelines for normal tissue sparing^26^, similar templates were created for high-risk prostate and endometrial cancer cases.

### Plan evaluation and analysis

Following the suggestions of Leung *et al*. paper for plan comparison^27^, several dosimetric parameters were used to build three metrics able to supply a global evaluation^1^: a healthy tissue conformity index (H) to describe the overall plan conformity^2^, a merit function (M) to describe the targets coverage and^3^ a penalty function (P) to evaluate the sparing of critical organs.

### Healthy tissue conformity index (H)

This index was defined as

$$H=\frac{1}{r}\cdot \mathop{\sum }\limits_{i=1}^{n}(\frac{T{V}_{RI,i}}{{V}_{RI,i}})$$

where r is the number of PTVs of different prescription dose, TV_RI_ is the target volume covered by the reference isodose and V_RI_ is the volume of the reference isodose cloud. Reference isodoses were set at 95% of each prescription dose level.

For example, for the two PTVs in high-risk prostate case, the equation is expanded as:

$$H=\frac{1}{2}\cdot ({H}_{1}+{H}_{2})=\frac{1}{2}\cdot [(\frac{T{V}_{95 \% ,PTV1}}{{V}_{95 \% ,PTV1}})+(\frac{T{V}_{95 \% ,PTV2}}{{V}_{95 \% ,PTV2}})]$$

### Target coverage index (M)

This index was defined as

$$M=\frac{1}{r}\cdot \mathop{\sum }\limits_{j=1}^{r}[\frac{\mathop{\sum }\limits_{i=1}^{p}(\frac{{V}_{Tj,Di}}{{V}_{Tj,RDi}})+\mathop{\sum }\limits_{i=1}^{q}(1-\frac{{V}_{Tj,Di}}{{V}_{Tj,ADi}})}{\mathop{\sum }\limits_{i=1}^{p}(\frac{100}{{V}_{Tj,RDi}})+q}]$$

where r is the number of targets of different prescription dose, p is the number of cold spot checks, q is the number of hot spot checks, V_Tj,Di_ is the volume of the jth target in % receiving a dose of at least the ith dose level, V_Tj_, R_Di_ is the minimum volume of the jth target in % receiving at least the ith dose level and V_Tj,ADi_ is the allowable volume of the jth target in % receiving at least the ith dose level.

For example, for the two PTVs objectives in high-risk prostate case as reported in Table 1, the equation is expanded as:

$$M=\frac{1}{2}\cdot ({M}_{1}+{M}_{2})=\frac{1}{2}\cdot \{\frac{[\frac{{V}_{PTV1,95}}{98}+\frac{{V}_{PTV1,98}}{95}]+[1-\frac{{V}_{PTV1,107}}{5}]}{[\frac{100}{98}+\frac{100}{95}]+1}+\frac{[\frac{{V}_{PTV2,95}}{98}+\frac{{V}_{PTV2,98}}{95}]+[1-\frac{{V}_{PTV2,107}}{5}]}{[\frac{100}{98}+\frac{100}{95}]+1}\}$$

Note that the denominators represents the maximum possible score.

### Normal tissue sparing index (P)

This index was defined as:

$$P=\frac{1}{n}\cdot \mathop{\sum }\limits_{j=1}^{n}[\frac{1}{m}\cdot \mathop{\sum }\limits_{i=1}^{m}(1-\frac{{V}_{Oj,Di}}{{V}_{Oj,ADi}})]$$

where n is the number of critical organs to be monitored, m is number of check points used for the jth critical organ, V_Oj,Di_ is the volume of the jth critical organ in % receiving a dose of at least the ith dose level and V_Oj,ADi_ is the allowable volume of that organ in % receiving at least the ith dose level.

For example, in the high-risk prostate case, P becomes

$$P=\frac{1}{4}[{P}_{rectum}+{P}_{bladder}+{P}_{bowel}+{P}_{femurs}]$$

where, for the rectum QUANTEC objectives reported in Table 1, the equation is expanded as:

$${P}_{rectum}=\frac{1}{3}\cdot [(1-\frac{V50}{50})+(1-\frac{V60}{35})+(1-\frac{V65}{25})]$$

and similarly for the other critical structures.

Main relevant OARs for the determination of P index were the rectum, the bladder, the small bowel and the femurs for prostate and endometrial cases, and PRV_spinal cord, PRV_brainstem, PRV_optic chiasm, parotid glands, lens and pharyngeal constrictor muscles for head-neck cases.

### Plan quality index (PQI)

A comprehensive Plan Quality Index (PQI) can be formulated consolidating the three different metrics H, M and P into a single figure obtained using the following Euclidean distance between the points (H,M,P) and (1,1,1).

$$PQI=\sqrt{{(1-H)}^{2}+{(1-M)}^{2}+{(1-P)}^{2}}$$

This index represents the overall quality of a treatment plan. This choice was made because it was considered the most appropriate to represent the plan quality deviation from the ideal case. i.e. the point (0,0,0). This must be interpreted as how far a plan is away from perfection, i.e. H = 1, M = 1, P = 1 or (1,1,1). So, for an ideal case, PQI = 0 while for the worst scenario is PQI = √3.

In order to compare previous values with more common indices we calculated the conformation numbers (CNs) for each target volume as suggested by the Van’t Riet *et al*.^28^:

$$CN=\frac{T{V}_{RI}}{TV}\times \frac{T{V}_{RI}}{{V}_{RI}}$$

where TV_RI_ was the target volume covered by the reference isodose, TV was the target volume, and V_RI_ was the volume of the reference isodose. The first part of this equation defines the quality of target coverage and the second part defines the volume of healthy tissues receiving a dose greater than or equal to the prescribed dose. CN ranges from 0 (complete PTV geographic miss) to the ideal value 1 (perfect conformity of the reference isodose to the PTV). Reference isodose was selected as 95% of the prescribed dose. Note that the second part of this equation represents the H value for each target.

Last, the integral dose (ID) received by non-tumour tissues was calculated as the product between mean dose and non-tumour tissue volume (Gy ∙ cc).

Differences between manual and automated plans were quantified using the Wilcoxon matched-pair signed rank with a statistical significance at p < 0.05.

### Plans variability

To evaluate the variability between MP and AP plans, we first calculated the coefficient of quartile variation (CQV) of the aforementioned metrics (CNs, H, M, P and PQI) for each of the three anatomical site. CQV was defined as the ratio between the difference and the sum of first and third quartiles and was adopted because of its statistical robustness when dealing with data with outliers and/or skewed distributions^29^.

Then, in order to quantify the differences in standard deviations (SDs) of CNs, H, M, P and PQI metrics we performed the Levene’s test for homogeneity of SDs when data comes from non normal distributions, with statistical significance at p < 0.05.

### Planning and treatment efficiency

For all patients, the total planning time (human inputs, optimization loops and dose calculation times) and the total number of monitor units were recorded for both MP and AP plans; all optimization processes were performed on a local server (HP Z800 workstation, 2.80 GHz).

### Dosimetric verification

Dose distributions were measured utilizing the 1500 2D ion-chamber array together with the Octavius-4D phantom^30^ both developed by PTW (PTW, Freiburg, Germany). The 1500 2D-array consists of a matrix of 1405 ion chambers with a size of 4.4 mm × 4.4 mm × 3.0 mm. This array is inserted into the Octavius-4D motorized cylindrical polystyrene phantom, capable to rotate synchronously with the gantry, so that the beam always hits the array in a perpendicular way, then allowing the possibility of 3-dimensional dose reconstruction and comparison. Measured and calculated dose distributions were compared by means of the gamma evaluation, based on the theoretical concept introduced by Low *et al*.^31^. Following the recent suggestions of the AAPM report No. 218^32^, dosimetric verification was considered optimal if the percentage of points fulfilling gamma index criteria exceeded 95% using 3% for dose criterion (global) and 2 mm for the distance to agreement criterion.

### Physician’s plan scoring

Two senior radiation oncologists independently performed a blind clinical evaluation of all AP and MP plans, based on the dose distributions, DVHs for all structures and a summary table reporting the most important parameters. The radiation oncologists rated the plans at first judging the clinical acceptability of each plan (pass or not pass) and secondary expressing their preferences using a clinical judgement based on a three-score scale (AP better than MP, MP better than AP and no preference). Cohen’s kappa coefficient k was calculated to assess the inter-clinicians agreement^33^, with score defined as excellent (k > 0.81), good (0.61 < k < 0.80), moderate (0.41 < k < 0.60), fair (0.21 < k < 40) and poor (k < 0.20).

## Results

### Head-neck cancer cases

**Figure 2:**
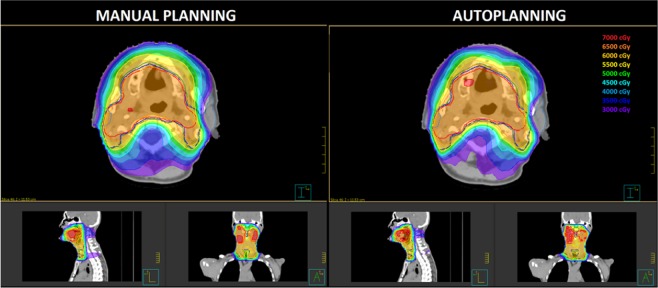
Comparison of dose distribution in axial, sagittal and coronal planes for a representative patient. Isodose curves are shown from 30 Gy to 70 Gy in 5 Gy steps. The PTV1, PTV2 and PTV3 target volumes are shown in red, blue and green contours, respectively.

A summary of H, M and P indexes for MP and AP plans is reported in Table 2. Global PQI scores for MP and AP plans were found to be 0.796 ± 0.059 and 0.722 ± 0.056, respectively. No significant differences were found for the target coverage M index for nodal target volumes (PTV2 and PTV3) but MP plans showed larger hot-spot regions inside the PTV1 providing a worse M value. H index values show a higher capability of AP plans to better conform the doses to target volumes, especially to the prophylactic volumes. Regarding P index values for organs at risk sparing, AP plans provided a major sparing of parotid glands and a reduction of mean dose of 10% (3.7 Gy, p = 0.022). Wilcoxon test also showed that the integral dose delivered to the patient body was significantly lower for AP plans than for the MP plans, with a reduction of 6.6% (p = 0.003). Figure 2 shows the dose distributions comparison for a representative patient.

**Table 2:** Comparison of scoring metrics between manual and automated planning for head-neck tumor cases.

	Manual Planning (MP)		Automated Planning (AP)		p _Wilcoxon_
	Mean	Range (min-max)	Mean	Range (min-max)	
**Conformation numbers**					
CN 1	0.565	0.297–0.756	0.633	0.381–0.783	0.006
CN 2	0.572	0.234–0.722	0.602	0.305–0.713	0.012
CN 3	0.645	0.555–0.712	0.695	0.656–0.737	0.006
**Conformity Index of Healthy Tissue (H)**					
H_PTV1_	0.576	0.300–0.787	0.643	0.376–0.801	0.006
H_PTV2_	0.587	0.236–0.756	0.623	0.308–0.777	0.004
H_PTV3_	0.677	0.576–0.733	0.719	0.661–0.791	0.004
H	0.614	0.480–0.739	0.662	0.579–0.756	0.003
**Target Coverage (M)**					
M_PTV1_	0.815	0.634–0.927	0.946	0.909–0.997	0.003
M_PTV2_	0.954	0.922–0.983	0.956	0.924–0.981	0.790
M_PTV3_	0.934	0.871–0.970	0.939	0.885–0.983	0.477
M	0.901	0.854–0.937	0.947	0.912–0.968	0.004
**Normal Tissue Sparing (P)**					
P_parotids_	−0.161	−0.752–0.144	−0.039	−0.683–0.141	0.008
P_lens_	0.554	0.329–0.692	0.573	0.347–0.766	0.824
P_PRV spine_	0.216	0.146–0.328	0.242	0.194–0.324	0.026
P_PRV brainstem_	0.329	0.231–0.478	0.422	0.261–0.709	0.013
P_PRV optic chiasm_	0.917	0.829–0.965	0.921	0.866–0.959	0.477
P_pharyngeal const. muscles_	0.045	−0.038–0.184	0.048	−0.021–0.182	0.721
P	0.316	0.157–0.373	0.368	0.183–0.452	0.003
Plan Quality Index (PQI)	0.796	0.739–0.909	0.722	0.640–0.858	0.003
Integral Dose (Gy*cc*10^5^)	1.079	0.615–1.528	1.008	0.586–1.374	0.003

### High-risk prostate cancer cases

**Figure 3:**
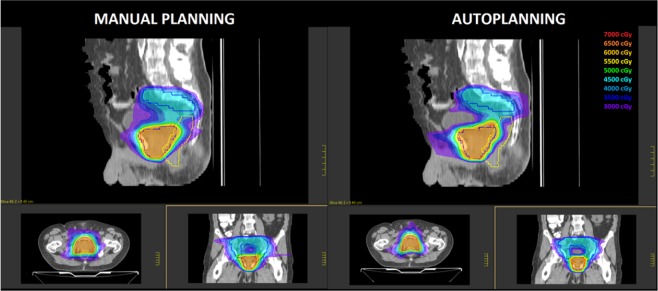
Comparison of dose distribution in axial, sagittal and coronal planes for a representative patient. Isodose curves are shown from 30 Gy to 60 Gy in 5 Gy steps. The PTV1 and PTV2 target volumes are shown in red and blue contours, respectively.

A summary of H, M and P indexes for MP and AP plans is reported in Table 3. Global PQI scores for MP and AP plans were found to be 0.429 ± 0.053 and 0.400 ± 0.0.049, respectively. No significant differences were found for the target coverage M index alone. H conformity index values was significantly better for AP plans (p = 0.003) suggesting an higher capability of dose conformation to the large concave shaped nodal target volume, as shown graphically in Fig. 3 for a representative patient. This ability also translated into a significant reduction of integral dose to non-tumour volumes of 7.2%. Regarding P values for OARs sparing, no significant differences were found for all relevant OARs between the two techniques (p = 0.477).

**Table 3:** Comparison of scoring metrics between manual and automated planning for high-risk prostate cancer cases.

	Manual Planning (MP)		Automated Planning (AP)		p _Wilcoxon_
	Mean	Range (min-max)	Mean	Range (min-max)	
**Conformation numbers**					
CN 1	0.820	0.754–0.866	0.821	0.767–0.864	0.722
CN 2	0.604	0.516–0.712	0.677	0.652–0.742	0.003
**Conformity Index of Healthy Tissue (H)**					
H_PTV1_	0.835	0.759–0.884	0.850	0.745–0.940	0.062
H_PTV2_	0.618	0.585–0.666	0.685	0.637–0.748	0.003
H	0.726	0.672–0.754	0.767	0.699–0.817	0.008
**Target Coverage (M)**					
M_PTV1_	0.959	0.843–0.986	0.968	0.913–0.995	0.878
M_PTV2_	0.943	0.925–0.964	0.941	0.913–0.970	0.721
M	0.951	0.900–0.975	0.954	0.913–0.981	0.878
**Normal Tissue Sparing (P)**					
P_rectum_	0.495	0.222–0.855	0.501	0.275–0.834	0.594
P_bladder_	0.641	0.250–0.893	0.651	0.267–0.997	0.859
P_femurs_	1,000	1.000–1.000	1,000	1.000–1.000	1.000
P_small bowel_	0.557	0.288–0.997	0.559	0.292–0.998	0.424
P	0.673	0.449–0.863	0.678	0.458–0.858	0.477
Plan Quality Index (PQI)	0.434	0.301–0.639	0.406	0.272–0.595	0.042
Integral Dose (Gy*cc) 10^5^	2.671	2.103–3.340	2.452	1.950–3.019	0.003

### Endometrial cancer cases

**Figure 4:**
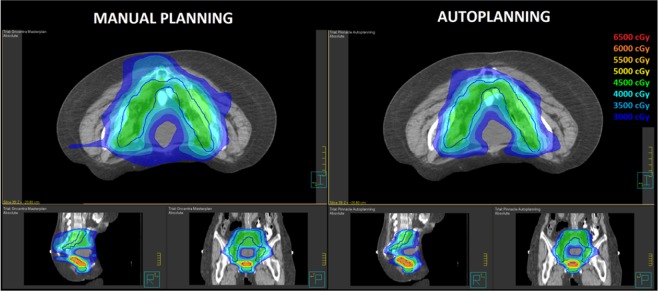
Comparison of dose distribution in axial, sagittal and coronal planes for a representative patient. Isodose curves are shown from 30 Gy to 60 Gy in 5 Gy steps. The PTV1 and PTV2 target volumes are shown in red and blue contours.

A summary of H, M and P indexes for MP and AP plans is reported in Table 4. Global PQI scores for MP and AP plans were found to be 0.532 ± 0.095 and 0.472 ± 0.081, respectively. As for prostate cases, while no significant differences were found for the target coverage M index, H values showed a higher capability of AP plans to better conform the dose distribution to target volumes, especially to the large concave nodal volumes. This capability is highlighted in Fig. 4 showing the dose distribution for a representative patient and it is also evidenced by a significant reduction in the integral dose to non-tumor tissues of 10% (p = 0.010). Regarding P values for OARs sparing, no significant differences were found for all relevant OARs between the two techniques (p = 0.953).

**Table 4:** Comparison of scoring metrics between manual and automated planning for endometrial cancer cases.

	Manual Planning (MP)		Automated Planning (AP)		p _Wilcoxon_
	Mean	Range (min-max)	Mean	Range (min-max)	
**Conformation numbers**					
CN 1	0.602	0.472–0.681	0.647	0.543–0.724	0.074
CN 2	0.604	0.538–0.704	0.702	0.665–0.743	0.005
**Conformity Index of Healthy Tissue (H)**					
H_PTV1_	0.604	0.472–0.683	0.645	0.543–0.724	0.139
H_PTV2_	0.602	0.559–0.691	0.725	0.690–0.781	0.008
H	0.603	0.540–0.687	0.685	0.617–0.744	0.011
**Target Coverage (M)**					
M_PTV1_	0.979	0.917–0.999	0.985	0.958–0.998	0.374
M_PTV2_	0.961	0.927–0.988	0.960	0.928–0.984	0.515
M	0.970	0.943–0.994	0.972	0.961–0.988	0.515
**Normal Tissue Sparing (P)**					
P_rectum_	0.737	0.625–0.884	0.739	0.657–0.874	0.859
P_bladder_	0.891	0.767–0.981	0.895	0.760–0.958	0.767
P_femurs_	0.894	0.870–1.000	1.000	1.000–1.000	0.180
P_small bowel_	0.490	0.125–0.746	0.484	0.104–0.723	0.374
P	0.647	0.446–0.781	0.650	0.441–0.766	0.953
Plan Quality Index (PQI)	0.532	0.425–0.668	0.472	0.385–0.651	0.015
Integral Dose (Gy*cc*10^5^)	3.196	2.392–4.468	2,881	2.111–3.999	0.010

### Plans variability comparison

**Figure 5:**
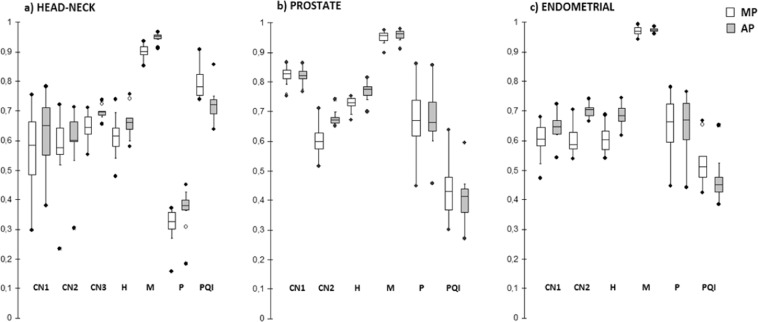
Whiskers box-plots of CNs, H, M, P and global PQI for the three anatomical sites for both MP and AP plans. The central line marks the median, the edge of the box are the 25^th^ and 75^th^ percentiles, black circles represent the extreme values. The whiskers extend to the adjacent values. The extent of the boxes represent the Inter Quartile Range (IQR).

Figure 5 shows the whiskers box-plots of CNs, H, M, P and global PQI for the three anatomical sites. In particular, 1 out of 36 patients (an high-risk prostate patient) reported a worse PQI value for AP plans. The figure also shows a qualitative reduction of CQV values for plans optimized with AP technique for almost all metrics. Table 5 reports the CQV and SD values calculated for the principal metrics used for plan comparison. AP plans reported a narrowing of the variability range of CQV for the global PQI values from 21% for endometrial cases to 37% for head neck cases. For each metric, the results of Levene’s test for homogeneity of SD between MP and AP plans are reported. In particular, AP plans reported a significant decrease in plans variability for the conformation numbers related to dose conformity to the large concave and irregular lymph-nodal volumes (CN2 and CN3).

**Table 5:** Summary of Coefficients of Quartile Variations (CQV) and standard deviations (SD) of CNs, H, M, P and global PQI for the three anatomical sites.

Metric	MP		AP		p levene
	CQV	S D	CQV	S D	
Head-neck cases					
CN1	0.156	0.148	0.126	0.123	0.537
CN2	0.074	0.129	0.053	0.112	0.813
CN3	0.045	0.047	0.007	0.021	0.034
H	0.052	0.076	0.029	0.056	0.496
M	0.015	0.027	0.006	0.018	0.299
P	0.084	0.075	0.043	0.066	0.902
PQI	0.044	0.059	0.032	0.056	0.604
Prostate cases					
CN1	0.017	0.036	0.015	0.030	0.546
CN2	0.047	0.061	0.014	0.025	0.042
H	0.015	0.031	0.019	0.025	0.567
M	0.012	0.020	0.011	0.019	0.832
P	0.089	0.118	0.071	0.106	0.672
PQI	0.132	0.098	0.099	0.074	0.511
Endometrial cases					
CN1	0.053	0.067	0.035	0.053	0.510
CN2	0.048	0.053	0.018	0.022	0.024
H	0.053	0.050	0.031	0.040	0.600
M	0.011	0.017	0.004	0.008	0.167
P	0.098	0.116	0.091	0.107	0.801
PQI	0.068	0.095	0.056	0.081	0.682

For each metric, the results of Levene’s test for homogeneity of SD between MP and AP plans are reported.

### Treatment efficiency and dosimetric verification

Table 6 reports a summary of the treatment planning efficiency and the delivery metrics. Average total treatment planning time was about 60 minutes for AP plans in high-risk prostate and endometrial cases and 80 minutes for head-neck cases. Compared to MP plans, a significant larger number of monitor units was observed for AP plans, especially for head-neck cases, reflecting an increased level of fluence modulation.

**Table 6:** Overview of treatment delivery metrics.

	MP		AP		p
	Mean	STD	Mean	STD	
Head-neck cancer cases					
MUs	508	44	586	39	0.003
Planning time (minutes)	188	44	82	6	0.000
Beam-on-time (minutes)	2.3	0.2	2.4	0.2	0.133
γ pass-rate (%)	97.9	1.4	97.8	1.4	0.749
High-risk prostate cancer cases					
MUs	528	58	572	45	0.003
Planning time (minutes)	180	40	61	4	0.000
Beam-on-time (minutes)	2.2	0.2	2.3	0.2	0.349
γ pass-rate (%)	98.3	1.4	98.3	1.5	0.811
Endometrial cancer cases					
MUs	540	122	583	78	0.015
Planning time (minutes)	175	41	60	4	0.000
Beam-on-time (minutes)	2.1	0.2	2.2	0.2	0.286
γ pass-rate (%)	98.5	1.4	98.3	1.4	0.983

Pre-treatment verification was performed for all plans. With criteria equal to 3%(global) −2 mm for γ-index, the average pass-rate was 98.2 ± 1.4% for MP plans and 98.1 ± 1.4% for AP plans (p = 0.882).

### Physician’s plan scoring

Cohen’s kappa coefficient resulted in a intra-observer variability equal to 0.83 indicating an almost perfect agreement. Regarding the assessment of all plans by the two radiation oncologists, the clinical score of AP plans was equal or better than MP plans in 97% and 94% of cases for both clinicians, respectively. The clinical evaluation of plan quality was favourable to AP plans for both radiation oncologists.

## Discussion

In the present study we explored the potential of a fully template-based automated VMAT planning engine implemented in Pinnacle TPS for challenging treatments executed in clinical routine. Head-neck, high-risk prostate and endometrial cancer sites were chosen because they involve large concave-shaped target volumes, multiple dose prescription, use of simultaneous integrated boost strategy and a large number of OARs adjacent or partially overlapping targets, then presenting the most complex and challenging problem for the plan optimization algorithms. The resulting plans were then compared with clinically accepted VMAT treatment plans generated by experienced medical physicists. The selection of optimal plans from different competing techniques or planning strategies has always been a daunting task, relying on dose volume histogram metrics and visual inspection of isodose distributions, often providing ambiguous evaluations. For this reason, a few quantitative indexes have been introduced to quantitatively describe the quality of a given plan^27,34^. In particular, Leung *et al*. proposed a new dose-volume based index for intensity-modulated plans called Plan Quality Index, able to simultaneously describe the overall plan conformity, the target coverage and the doses to critical organs^27^. The authors reported that this index improved the plan quality discerning power with respect to conventional comparison strategies. Following the suggestions of Leung *et al*. paper, we adopted the proposed PQI as fundamental metric to determine plan quality and for plan comparison purposes. Our results show that for all the anatomic sites, AP plans were able to provide similar, and in some cases better, plan quality of MP plans. AP plans significantly improved dose conformity, especially to large concave nodal target volumes, in all anatomical sites, but no statistically significant differences were found in terms of targets coverage. Similarly, no statistically significant difference were found for all relevant OARs dose sparing in high-risk prostate and endometrial cancer cases. However, AP plans showed an improvement in OARs sparing in head-neck cancer cases, i.e. in cases with the most complex anatomic scenario. In this case, maximal dose to PRV brainstem was reduced on average by 4.3 Gy and parotids mean dose was reduced on average by 3.7 Gy, that may be clinically relevant to reduce xerostomia.

These dosimetric findings were confirmed by the two radiation oncologist in the blind clinical evaluation session who considered AP plans better or equal to MP plans in more than 90% of cases.

A potential bias of this kind of planning comparison studies is that the quality of MP plans should be as high as possible (poor quality of MP plans obviously would favour AP plans). In our case, all clinically MP plans were created by two medical physicist with 10 years experience in VMAT planning, with the aim to obtain not only high quality plans but also a reduction of interplanner variability. As reported in Table 6, AP plans achieved a reduction of variability expressed by the CQV metrics for almost all dosimetric metrics for the three anatomical sites, with statistical significance for dose conformity. AP engine not only significantly improved dose conformity for complicated target geometry (including nodal involvement) but it has also the potential to drive a reduction of human-caused variability in VMAT planning for conformal coverage and dose distributions.

It must be highlighted that manual planning for complex cases is a challenging task, based mainly on the planner experience. Planners, although very experienced, never “a priori” know how much a plan can be optimized nor they can ensure that all dosimetric constraints on all OARs have been tightened as much as possible. This result has also been observed in other recent studies^5,15^ focused on the optimization of prostate treatment with different automated algorithms as Rapidplan and Erasmus-Icycle. From this point of view, automated planning could allow more consistent outcomes in treatment planning studies and clinical trials thanks to their greater ability to reduce the inter- and intra-planner variations.

Regarding planning and treatment efficiency, AP plans resulted in 8–15% increase of MUs, a result in agreement with other experiences with Autoplanning engine^22^, and suggesting an increase of plan complexity and fluence modulation. However, the MUs increase did not translate in a lower pass rate during pre-treatment verification on the Octavius-4D phantom, which resulted in strong agreement with MP plans pass rate (p = 0.882). Moreover, unlike expected, the increase of MUs number did not increase the integral dose to the patient, which was lower by 6.6–10.1%, theoretically reducing the risk of secondary malignancies^35^.

Mean overall planning time including human inputs, optimization loop processes and calculation times was 60 minutes and 80 minutes for pelvic and head-neck AP plans, respectively (about a third of time needed for manual planning).

Perhaps the most important feature of the Autoplanning module is its ability, according to the vendor, to push the OARs dose sparing beyond the constraints specified in the Technique, towards physical achievable limits. This feature is unique and represents a significant change compared to other dosimetric planning engines or to the natural human planning strategy, in which the primary goal is the achievement of objectives judged to be clinically effective. In the present paper, this ability was reported in the treatments of the head-neck district, where AP plans showed a significant reduction in the average dose to the parotid glands of about 10% and of the maximum dose to brainstem and spinal cord, well beyond the objectives that had been assigned in the Technique. Clearly, to definitively prove the aforementioned claim it would be necessary to demonstrate that AP plans are Pareto optimal, i.e. one or more objectives (as OARs sparing) cannot be improved without worsening at least one other (as target coverage). This demonstration is a challenging mathematical task^36^ and is beyond the scope of the present paper.

An advantage of Autoplanning with respect to other strategies for automatic planning based on KBP knowledge-based approach is that it does not rely on a database of prior patients. This database must usually be filled with a large number of high quality plans for each protocol and disease site, whose clinical implementation translated in a labor-intensive process. Any changes in contouring protocol or dose prescription or planning techniques could require the generation of a new database. On contrary, in our experience only a small set of training patients for each anatomical site (five patients in our experience) was necessary as starting point for the implementation of the Techniques in Autoplanning by an expert team of medical physicists.

However, in the case of head-neck cancer site, we faced the problem of high point doses in serial OARs as the spinal cord or brainstem when they lie very close or partially inside the PTV. These cases have been solved with a further manual tuning of dose objectives in the post optimization so as to decrease the dose to these serial OARs below the acceptable values. This re-optimization step does not require more than 15 minutes of dose calculation time. From this point of view, AP strategy can always be considered a high quality starting point for further plan optimization, that is a tool able to increase the overall quality of planning, rather than a tool that could completely remove the need of manual optimization.

A potential limitation of the PQI evaluation method is that different combinations of H, M and P values can provide identical PQI values when comparing two plans (i.e. one plan may have a better M while another plan may have a better P). In this scenario, clinicians would inevitably decide which one would benefit the patient most, focusing attention on the coverage of the targets rather than on dose conformity or OARs sparing. Moreover, the actual H, M and P indexes are defined so that each dosimetric parameter has the same weight, while, in some specific clinical cases, clinicians may prefer that an objective for an OAR or a target have more weight than another.

Two additional potential benefits of Autoplanning template-based module are currently under investigation. The first one is the feasibility of rapid and easy knowledge-sharing between different institutions. The In our experience, for high-risk prostate and endometrial treatments, Autoplanning normally created optimal plans in a “one-button click” procedure without any planner intervention for manual tuning. Techniques for specific anatomical sites can be successfully shared and implemented across multiple centres with simple adaptations to local protocols, allowing each centre to obtain optimal plans with the same quality^37^. The second benefit concerns the use of Autoplanning in the adaptive radiotherapy setting. In this case, the goal to correct for daily tumour and normal tissue variations through modification of original plan is hampered by the time-consuming re-planning process, representing nowadays the major obstacle for large scale implementation of this strategy. Improvement in Autoplanning, therefore, has the potential to make routine online adaptive radiotherapy a possibility^38^.

## Conclusion

We evaluated the Pinnacle Autoplanning engine to be a robust clinical tool, reporting significant increase of dose conformity with respect to manual planning. The blinded clinical scoring confirmed the dosimetric results, showing that in more than 90% of the evaluations AP plans were judged of equal or better quality with respect to MP plans. The reductions of plans variability and overall treatment time suggest the use of Autoplanning as a valuable tool to standardize high plan quality and improve clinic efficiency. Owing to dosimetric and clinical advantages, Autoplanning engine is an effective device enabling the generation of VMAT high quality treatment plans according to institutional specific planning protocols.

Future studies are needed to expand Autoplanning to other treatment techniques such as extracranial stereotactic radiotherapy.
